# Impacts of the Property Assessed Clean Energy (PACE) program on the economy of California^☆^

**DOI:** 10.1016/j.enpol.2019.111087

**Published:** 2020-02

**Authors:** Adam Rose, Dan Wei

**Affiliations:** a Schwarzenegger Institute for State and Global Policy, Sol Price School of Public Policy, 650 Childs Way, RGL 230, University of Southern California, Los Angeles, CA, 90089, USA; b Schwarzenegger Institute for State and Global Policy, Sol Price School of Public Policy, 100D University Gateway, 3335 S Figueroa St., University of Southern California, Los Angeles, CA, 90007, USA

**Keywords:** Property assessed clean energy, Economic impacts, Energy efficiency, Renewable energy, Water conservation, Incentives

## Abstract

The Property Assessed Clean Energy (PACE) Program is an innovative financing approach to meeting environmental goals. PACE financing is structured as an assessment to the property and paid along with the property tax bill. In addition to the direct environmental benefits, it also yields co-benefits of enhanced economic output and employment. This paper estimates the economic impacts of PACE in California by one of its major financing companies. These impacts include direct spending on structural improvements, reduction in spending on centralized power and water services, reallocation of spending from energy and water bills savings, and solar investment tax credits, among others. It also includes general equilibrium effects of these various factors. Our results indicate PACE financing yields sizable economic benefits. At the same time, the increased economic activity results in increased energy and water use that partially offsets some of the direct environmental gains. Furthermore, PACE has been subject to criticism because it gives financing companies the first lien on mortgages and because of anecdotal examples of some customers being lured by unscrupulous contractors. The direct environmental benefits and economic co-benefits of PACE should be factored into the policy debate over whether the Program should be further expanded or regulated.

## Highlights

• Property Assessed Clean Energy provides innovative financing for environmental goals.
• Ygrene Energy Fund has financed over 32,000 CA projects since 2013 for $760 million.
• Reduced net electricity use by 3.53 million MWh and natural gas use by 2.02 bcf in CA.
• Increased the NPV of GDP by $661 million and employment by 9,774 person-years in CA.
• Economic co-benefits should be considered in discussions of regulation and expansion.

## 1 Introduction

Property Assessed Clean Energy (PACE) is a way to finance a wide range of energy and water efficiency, renewable energy, and hazard reduction improvements permanently attached to residential and commercial properties. Established by state statutes and enabled by local governments, PACE financing, unlike traditional alternatives, is an assessment to the property and not the property owner. Only certain types of improvements are eligible to be financed through PACE, and all PACE programs are required to provide public benefits in the host state. PACE provides one hundred percent of the cost of qualified structural improvements, which the property owner repays annually or semi-annually through a special assessment added to the property tax bill.

The direct benefits of PACE financing are rather straightforward—reduction in energy and water use, reduction in pollutants associated with this use, and reduction in hazard vulnerability. However, the financing also generates economic *co-benefits* directly and indirectly. These benefits are more complex than those measured in ordinary economic impact analysis associated with typical economic stimuli such as the opening of a new factory or mine. Such stimuli, as well as PACE financing, all generate direct impacts on site and multiplier or broader general equilibrium impacts throughout the rest of the economy. However, many conventional stimuli provide products primarily for export and do not interact much with the host economy beyond a limited number of supply chain and wage/salary income increases and spending. PACE improvements, on the other hand, provide energy and water cost savings that stimulate spending on consumer goods and enhance business profits. At the same time, the efficiency improvements reduce the production of conventional energy and centralized water services and affect their prices. Hazard mitigation improvements help prevent the need to dip into savings to repair damaged structures and compensate for business interruption.

This study performs a regional economic impact analysis of PACE financing by one of the leading firms in the market—Ygrene Energy Fund, Inc. Since 2013, and through the end of July 2018, Ygrene has provided more than $1.16 billion to finance over 54,500 property improvement projects in over 500 cities and counties in California, Florida, and Missouri (Ygrene, 2018). We estimate the net impact of these financing projects on the economy of one of the major states in which Ygrene does business – California. We focus on major macroeconomic indicators of gross output (sales revenue), gross domestic product (GDP), personal income, and employment. The estimation methodology includes both direct and various types of indirect effects rippling through the supply chains of the economy. The approach is that of Economic Consequence Analysis (ECA), which utilizes most of the principles of Benefit-Cost Analysis (BCA) at the multi-market level, but focuses on macroeconomic indicators rather than the traditional welfare (personal “well-being”) measures (see, e.g., Rose et al., 2011; Rose et al., 2017; Farrow and Rose, 2018).

This paper fills an important void in the literature on the total regional economic, environmental, and resource use impacts of PACE financing. Most of the analyses of PACE financing have dealt with its strengths and limitations (see, e.g., Goodman and Zhu, 2016; Schreiber and Cisar, 2018, as well as the more detailed discussion of these issues in Section IX below relating to policy implications). Two major studies have been performed on the extent to which PACE financing has stimulated the adoption of solar energy. Kirkpatrick and Bennear (2014) undertook an econometric analysis and found that PACE financing resulted in a 108% increase in the adoption of rooftop solar technology in California. On the other hand, Deason and Murphy (2018) concluded that this effect was only somewhere between 7% and 12% in the state. Only one study to date has examined the broader economic impacts of PACE financing, and that study was performed very early in the history of the PACE Program. ECONorthwest (2011) utilized an input-output (I–O) model, a much simpler modeling approach than the macroeconometric model used in our study. That study examined the potential economic impacts of PACE financing in four US cities, and concluded that $4 million in program financing would result in $10 million of increased gross output (regional sales revenue). This implicit multiplier is significantly larger than the outcome of our analysis, and is probably due to the limitations of I–O modeling, especially its restrictive linearity. To date no other studies have been performed of the potential of PACE financing to lower greenhouse gas emissions or water use as well.

## 2 Contributions of PACE financing

The overall purpose of PACE financing is to provide funds for home and business building improvements that directly produce environmental or hazard risk reduction benefits. Additionally, PACE can provide financing to those that might otherwise have difficulty securing other forms of credit. In particular, PACE financing is not a credit score-dependent product, in part because the funds are collateralized by a first lien on the property, as well as a variety of underwriting requirements set forth by state and local governments. Another direct benefit to consumers and small businesses is that there is no down payment or upfront cost to the borrower.^1^ Also, terms of the assessment are directly tied to the useful life of the improvements and thus can extend beyond typical terms of traditional financing, although earlier repayment options are available.

The direct benefits of PACE financing analyzed in this study include:

• Decreases in electricity and natural gas use
• Increase in renewable electricity generation
• Decreases in the emission of greenhouse gases
• Decreases in water use
• Decreases in vulnerability to earthquakes

These benefits are measured in terms of physical units, as well as dollar values where possible. Overall, they result in improvements in the efficiency of the economy by eliminating wasteful practices relating to water and energy use and also to the cost to society of “externalities” such as pollution. In addition to the “efficiency-improvement” contributions of PACE financing, there is also an improvement in equity, or fairness, in society because the PACE Program is available equally across all property-owner income groups and small businesses.

In addition, the “co-benefits” of PACE financing analyzed in the study are:

• Increase in business sales revenue, GDP, personal income, and employment
• Increase in tax revenues for various levels of government
• Decreases in property damage
• Decreases in disaster relocation cost

These co-benefits, together with other types of co-benefits (such as decreases in ordinary air pollutants and improvement in public health) that are not quantified in this study, add to the “business case”, “household benefits” and “overall societal well-being” of PACE financing.

Between its start in 2013 through July 2018, Ygrene financed 31,867 residential properties and 646 commercial properties in California.^2^ Table 1 presents the distribution of the total of $0.76 billion contract dollars among the various improvement categories in California. It covers the period 2013 to mid-2018, and nine categories of improvements, eight of which relate to reductions in GHG emissions or water use. Note that over 92% of PACE funds are for energy efficiency upgrades and renewable energy installations. Also note the general upward trend on financing over time.

**Table 1:** Ygrene PACE Financing in California by Improvement Category (in thousands of 2015$).

	2013	2014	2015	2016	2017	2018	Total
Building Envelope Energy Efficiency	$259	$2,782	$18,718	$57,171	$68,486	$22,405	$169,821
Solar	$802	$7,904	$40,024	$151,942	$77,342	$25,537	$303,552
Energy-Efficient Windows and Doors	$104	$1,322	$10,357	$32,767	$35,132	$9,420	$89,102
HVAC Efficiency	$180	$2,437	$26,083	$55,680	$32,828	$9,919	$127,126
Water Conservation	$36	$533	$4,393	$17,170	$25,276	$9,505	$56,913
Lighting Efficiency	$5	$75	$631	$2,263	$4,576	$1,208	$8,758
High-Efficiency Water Heating	$0	$0	$0	$171	$1,934	$709	$2,814
Earthquake Mitigation	$0	$0	$0	$0	$387	$975	$1,362
High-Efficiency Pool Equipment	$0	$0	$0	$30	$338	$132	$500
**Total**	**$1,386**	**$15,051**	**$100,207**	**$317,193**	**$246,300**	**$79,810**	**$759,947**

## 3 Economic analysis of PACE impacts

### 3.1 Direct economic impacts

Estimating the economic impacts of the PACE Program involves a number of considerations. It includes several positive stimulus factors, as well as some partially offsetting ones. Some of these impacts relate to the buildings in which improvements are made, while others relate to direct and indirect impacts off-site. Many of the benefits of the PACE Program pertain to improving the environment and are complex to assess because of the absence of market prices associated with them.

A set of positive stimulus factors that directly affect the economy stems from the purchase of materials and equipment, as well as the labor involved in their installation, for PACE improvements. Examples include energy-efficient water heaters, solar panels, windows, caulking material, and wages paid to workers with various skill levels. The demand for the various inputs (e.g., materials and equipment) into the PACE improvements directly increase the economic activities of several sectors that produce these inputs, which leads to further upstream and downstream interindustry multiplier effects. The demand also leads to the payment of wages/salaries to workers and returns to capital to business owners, which they subsequently spend, setting off further rounds of multiplier effects. Additional positive stimuli emanate from the operation and maintenance of PACE improvements throughout their useful life.

Another set of positive stimuli is associated with the savings from improved efficiency in the use of energy and water. Homeowners then use the major portion of these savings to purchase more of the typical basket of consumer goods, thereby generating additional economic activity. Business owners could use the savings for several purposes, including expanding production, lowering prices, or increasing wages and profits.

Similar savings stem from mitigation efforts that reduce vulnerability to disasters. Here, however, the assessment of spending is more complicated, because, in the absence of these improvements, property owners would spend money anyway to repair and reconstruct damaged facilities. On the surface this could be an equivalent amount to spending on the improvement and would appear to be a wash in terms of direct economic impacts as measured by standard economic indicators such as gross output or employment. However, implementing disaster mitigation improves the level of well-being of the property owners by reducing their risk of loss of property or income, while repair and reconstruction simply returns well-being to the original level. The well-being of homeowners and businesses are measured in this report in terms of their expenditures on hazard mitigation.

A set of direct substitution effects also needs to be taken into account, which offsets the aforementioned positive stimuli to a lesser, equal, or greater extent. Reduced spending on energy and water results in a decrease in the economic activity that produces them. The net effect is likely to be positive within most states, however, since the majority of the positive stimulus spending will increase the activity of in-state producers, while the inputs to displaced production are top-heavy with goods imported from other states (e.g., California imports nearly all of the natural gas used in its electric power plants).

Additional positive stimuli stem from interest and fees paid to Ygrene and from fees paid to state and local governments for operating costs, including permitting and inspection. Fees paid to governments at various levels increase their revenues and are likely to be spent on government provision of goods and services within a state, thus generating an additional direct stimulus. Payments to Ygrene increase its business activity, and hence wages and profits, with the former subsequently spent primarily within the state.

Yet another stimulus associated with PACE financing stems from the 30% solar energy federal investment tax credit (ITC). This payment essentially increases the disposable income of households and increases consumer expenditures on goods and services, on the one hand, and increases business profits, or possibly lowers prices, on the other. The credit is usually received by the property owners between 4 and 16 months after the installation of the solar energy system. We assume that the households will spend 50% of the credit dollars in the year of the installation, and 50% in the following year. Moreover, since the solar ITC is a federal tax incentive, we assume there will be no reduction in federal government spending in California or increase in other taxes in the state as an offset effect.

Finally, we summarize some additional benefits and downsides of PACE financing, the estimation of which are beyond the scope of this study. There is evidence that the financed improvements increase the value of the home or business, in part because of the lower energy and water costs, as well as reduced vulnerability to hazards. Goodman and Zhu (2016) estimated that, on average, homeowners that utilize PACE financing could recoup its value at the point-of-sale. Another type of benefit is the reduction in uncertainty in home operating costs, which could be estimated in several ways, though with some difficulty. One way is to consider the reduced amount of reserve funds that homeowners need to be set aside for the contingency of spikes in energy costs, for example. However, the reduced cost is not the reduced dollar value of the funds, but rather the carrying cost on them, which is only a few percentage points at most. Also, there is the likelihood that lower operating costs of a home due to energy and water savings and reduced hazard losses facilitate not only repayment of PACE financing but mortgage financing in general, thereby decreasing defaults. Some analysts have also pointed out that PACE improvements enhance the performance of a home, thereby yielding additional non-market benefits to its residents (Rocky Mountain Institute, 2017). See also the Policy Implications Section below.

A complex issue of the analysis is that the PACE Program provides assessments rather than outright grants. Since these assessments need to be repaid, this results in a diversion of expenditures from other goods and services by consumers or from production activities by businesses. Thus, with respect to the spending from and repayment of the assessments, there is a significant stimulus in the year(s) that the improvement is implemented, but a negative stimulus to the economy spread over the period during which the assessments are repaid. This repayment can come equivalently from prior savings or assets of the borrowers or from savings on the utility bills and insurance premiums that the improvement provides, or some combination of both.

Even the initial use of the assessments may involve some offsetting factors depending on the origin of the capital base of a PACE Program lender. The assessment of the PACE Program is being done at the local level within a state, so, if the capitalization of the lender comes from funds from out-of-state, then there is an equivalent positive stimulus from all the assessments. However, if the capital comes from within the state, we must evaluate whether it displaces the use of these investment funds for other purposes. If so, this displacement effect would have to be taken into account, and the ensuing direct and indirect impacts would have to be subtracted from the bottom-line impacts. The difficulty in addressing this component is that firms that invest in PACE securitizations are active in global markets. While many firms have headquarters in the US, some in states where PACE programs operate, we do not have access to their geographical investment portfolio. Thus, estimating where funds originated would be extremely difficult if not impossible. Given the relatively small amount of PACE financing as a proportion of total global capital market funds available, we believe that an assumption of zero in-state investment displacement is reasonable.

### 3.2 Direct energy and environmental impacts

A major set of direct impacts stems from reduced water use, reduced energy use, and reduced greenhouse gas emissions. Unfortunately, directly observable market prices do not exist for pollutants, so we will apply some measures in the literature of the damages they cause, where the benefits of PACE assessments represent these avoided losses. For example, we use the findings of a recent National Academies of Science report on the social cost of carbon (NRC, 2017)^3^; however, we are not able to estimate the value of reduced amounts of ordinary air pollution, though we posit that this is likely to be a small amount in comparison to carbon dioxide emissions. Similarly, we are not able to measure the value of any reduced amount of water pollution.

In the case of the benefits of decreased energy use and water use, we encounter two complexities. First, we could apply the value of these resources, but this would overstate the benefits because we have already counted them in the consumer savings. At the same time, simply using the values of these resources at market prices omits the consumer and producer surplus components and causes some underestimation. Hence, we simply present the amounts of energy and water savings in physical units.

### 3.3 Indirect economic impacts

As noted above, the spending on property improvements that are made possible by Ygrene financed local government assessments apply to the site of their application, or what is termed direct economic impacts. However, the estimation of the total, or macroeconomic, impacts includes the ripple, or multiplier, effects of the various increased or decreased spending streams, as well as the interaction of demand and supply in numerous markets. For example, the increased demand for high-efficiency water heaters directly stimulates the demand for inputs into their production, such as fabricated metals, insulation material, and labor. The process continues through a chain-reaction of indirect impacts, as more fabricated metal and insulation production stimulates demand for more of their inputs, and as the producers of these inputs demand more inputs, and so on.

The many types of linkages in the economy and macroeconomic impacts are extensive and cannot be traced by a simple set of calculations. They require the use of a sophisticated model that reflects the major structural features of an economy, the workings of its markets, and the interactions between them. In this study, we used the Regional Economic Models, Inc. (REMI), Policy Insight Plus (PI+) Model (REMI, 2018). This is the most widely used state and regional level macroeconometric modeling software package in the U.S. and has been extensively peer-reviewed. The REMI Model integrates key features of input-output (I–O) models, computable general equilibrium (CGE) models, econometric models, and the concepts of economic geography. A more detailed description of the REMI Model is presented in Section VI. The reader is also referred to Rose and Wei (2019) for a presentation of the structure and workings of the REMI Model.

## 4 Data

A summary of the data used in this study is presented in Appendix Table 1. Below we summarize each of the data sets and associated estimates of key parameters used in our modeling simulations.

Ygrene Energy Fund, Inc. provided us with extensive data on its financing from 2013 through July 2018. The data covered major characteristics of the individual assessments, including major assessment characteristics (e.g., type of improvement, useful life of the improvement, total contract amount, type of property, location, settlement date), financing characteristics (e.g., interest rates, amortization period, annual coupon, program fees, initial face amount), and characteristics of the property (e.g., building area, property value, mortgage amount, owner type).

The authors then mapped improvement expenditures to the 160 economic sectors in the REMI Model to calculate the indirect effects. This began by dividing these expenditures between sectors that produced/supplied the property improvement materials/equipment and installation of the equipment or retrofit of the structure.^4^

Data on energy and water savings were obtained from the “Ygrene Proprietary Impact Metrics Model” developed by Ygrene. This impact model is used to estimate the energy savings, water savings, natural gas savings, renewable energy generation, utility bill savings, and the associated greenhouse gas emissions reductions. The impact model was based on a 12-month data-set of Ygrene funded projects between July 2017 and June 2018. For each residential and commercial property in the data set, Baseline Energy Consumption by end-use (e.g., space heating, water heating, lighting, pool pumps) and Baseline Water Consumption by end-use (e.g., outdoor usage, toilet, faucet, shower) were determined to form the foundation upon which improvement level savings are estimated.^5^Once baseline usages were determined, savings potential was identified for 24 distinct improvement types,^6^ which roll into the 10 improvement categories listed in Table 1.

After the energy (electricity and natural gas) and water savings quantities were estimated at the improvement level, state-level energy and water rates for residential and commercial sectors and emissions factors were applied to these savings quantities to generate associated utility bill savings and greenhouse gas reductions.

All energy, water, utility bill, and greenhouse gas saving estimates were then divided by the total contract amount (cash equivalent of installed measures) within each of the improvement categories to derive the energy, water, utility bill, and greenhouse gas savings factors per thousand contract dollars by improvement type, state, and property type. These factors are then applied to the entire Ygrene portfolio based on the contract amounts for each improvement type, state, and property type to estimate annual savings.

To estimate lifetime savings, annual factors are applied for the useful life of each improvement type. When calculating lifetime utility bill savings, it is assumed that the prices of electricity, natural gas, and water will increase or decrease at the average annual historical growth rates over the past 6 years in each state (EIA, 2018a; EIA, 2018b; and Circle of Blue, 2018). Additionally, it is assumed that Solar PV output degrades at a constant annual rate over the lifetime of the panels, which affects lifetime energy, utility bill, and carbon emission savings.

Data relating to hazard mitigation improvements – reduction in risk from earthquakes– were obtained from various sources. Ygrene provided information on expenditures for these improvements. Hazard loss savings by borrowers from the installation of mitigation improvements were calculated by multiplying the expenditure data by the benefit-cost ratios (BCRs) for the earthquake threats for each improvement type. The BCRs were obtained from results calculated as part of the *Mitigation Saves 2* Study (Multihazard Mitigation Council (MMC), 2017; Porter, 2018). Note that these BCRs were not provided at the same level of detail as the improvement types, so it was necessary to aggregate the latter to a smaller number of categories.

Note also that due to the uncertainty surrounding some of the data, below we perform sensitivity tests around best estimates of data inputs into the analysis.

## 5 Direct benefits

### 5.1 Energy and environmental improvements

The implementation of Ygrene PACE improvements is estimated to result in substantial direct benefits from energy consumption reductions and water conservation. In California, the energy/water efficiency and renewable energy PACE projects are estimated to lead to electricity consumption reductions of 3.63 million megawatt hours (MWh), natural gas consumption reductions of 2.86 billion cubic feet (bcf), and water savings of 2.36 billion gallons over the entire useful life of the improvements. 7

### 5.2 Greenhouse gas emission savings

State-specific emissions factors were applied to the energy saving quantities to calculate the associated greenhouse gas reductions.^8^ In California, the Ygrene PACE improvements are estimated to result in GHG emission reductions of 1.15 metric MtCO2e.

### 5.3 Avoided disaster losses

We apply hazard reduction BCRs to the total contract dollar amounts of the projects to obtain estimates of the avoided disaster losses from the implementation of these disaster mitigation/resilience improvements (see, e.g., Rose, 2017). The results are that the $1.45 million investment in seismic retrofits and new home seismic improvements in California is estimated to result in $2.36 million of avoided property damage and $0.38 million avoided temporary relocation costs to homeowners (in 2015 dollars). The avoided disaster losses are calculated by adapting the benefit-cost ratios of disaster mitigation investment on hundreds of building improvements analyzed in the *Mitigation Saves 2* Study (Multihazard Mitigation Council (MMC), 2017). The MS2 study used a discount rate of 2.2% and a useful life of 50 years for retrofits to ordinary buildings. Additional unpublished data from MS2 were used to estimate the benefits of savings of relocation costs for homeowners during loss of services of their residences while they were being repaired or rebuilt. The avoided disaster losses are calculated for both residential and commercial buildings between 2013 and 2018 by multiplying the total PACE investment in seismic mitigation in these years by the corresponding BCRs, which ranged from 0.47 to 5.04 for commercial structures and from 0.27 to 2.92 for residential structures (see Rose and Wei, 2019).

## 6 REMI model simulation of indirect economic impacts

The REMI PI + Model was selected to evaluate the macroeconomic impacts (such as gross state output, employment, and personal income) of the PACE Program. It is the most widely used macroeconometric model to analyze the economic impact of energy and climate policies in the U.S. The REMI Model has evolved over the course of more than 30 years of refinement (see, e.g., Treyz, 1993). It is a packaged program, but is built with a combination of national and region-specific data. In addition to widespread use in the academic community, government agencies in practically every state in the U.S. have used a REMI Model for a variety of purposes, including evaluating the impacts of energy and/or environmental policy actions (REMI, 2019).

As a macroeconometric forecasting model, the REMI model covers the entire economy based on macroeconomic aggregate relationships such as consumption and investment. REMI differs somewhat in that it includes some key relationships, such as exports, in a bottom-up approach that allows evaluation of specific sector-based policy options. In fact, it makes use of the finely-grained sectoring detail of an input-output (I–O) model, i.e., it divides the economy into 160 sectors, and thereby depicts important distinctions among them.

The REMI model is able to analyze the quantity interactions between sectors (ordinary multiplier effects) but with refinements for price changes not found in I–O models. That is, the Model incorporates the responses of producers and consumers to price signals and the changes in other market and regulatory conditions, and captures the substitution effects and other price-quantity interactions. The REMI Model also brings into play features of labor and capital markets, as well as trade with other states or countries, including changes in competitiveness. The labor market in the REMI model is linked to a demographic module of population migration. It also includes input substitution between labor and other factors of production, market supply and demand, wage rate determination, and economic geography considerations of labor accessibility of individual industries.

The econometric feature of the REMI Model refers to two considerations. The first is that the model is based on inferential statistical estimation of key parameters based on pooled time series and cross-regional (panel) data. This gives the Model an additional capability of being able to extrapolate the future course of the economy, a capability that most other types of economic impact models usually lack.

The version of the REMI Model used includes two geographical regions: California and rest of U.S. The model is established based on U.S. and California historical data through 2016. However, in this study we primarily use the California region to simulate the economic impacts of PACE investment in that state.

Before undertaking any economic simulations, the estimates of the direct costs and savings of the PACE projects are translated to REMI PI + model inputs. This step involves the selection of appropriate economic activity and policy levers in the Model. Table 2 presents a summary of how direct effects are linked to various model “blocks” (modules) that contain these levers, as well as an indication of the direction of the impact of each of the direct impacts of PACE financing. The first two columns show the various direct costs incurred by and savings accruing to the business (commercial) sectors and the household (residential) sector. The third column presents the corresponding economic variables in the REMI PI + Model and their position within the Model (i.e., in which one of the five major blocks the policy variables are located). The last column indicates whether the impact represents a positive or negative stimulus to the economy.

**Table 2:** Linkages between direct costs/savings of PACE projects and REMI inputs.

Linkage	Direct Costs/Savings of the PACE Program		Policy Variable Selection in REMI	Positive or Negative Stimulus to the Economy
1	Upfront Spending on Property Upgrades/Retrofit – Construction/Installation		Output and Demand Block →Exogenous Final Demand (amount) for Construction sector → Increase	Positive
2	Upfront Spending on Property Upgrades/Retrofit – Building Materials/Components		Output and Demand Block →Exogenous Final Demand (amount) for products from multiple sectors → Increase	Positive
3	Expenditure on Ygrene Fees		Output and Demand Block →Exogenous Final Demand (amount) for Monetary Authorities, Credit Intermediation sector →Increase	Positive
4	Expenditure on Program Fees		Output and Demand Block →State and Local Government Spending →Increase	Positive
5	Interest Payment of PACE Assessments		Output and Demand Block →Exogenous Final Demand (amount) for Monetary Authorities, Credit Intermediation sector→Increase	Positive
6	Annual Amortized Payment by PACE Assessment Borrowers	Businesses (Commercial Sectors)	Compensation, Prices, and Costs Block →Capital Cost (amount) of Individual Commercial and Industrial Sectors →Increase	Negative
		Households (Residential Sector)	Output and Demand Block →Consumption Reallocation (amount) →All Consumption →Decrease	
7	Energy (Electricity and NG) and Water Savings	Businesses (Commercial Sectors)	Compensation, Prices, and Costs Block→ Production Cost of Individual Industrial and Commercial Sectors→Decrease	Positive
		Households (Residential Sector)	Output and Demand Block →Consumption Reallocation (amount) →All Consumption Sectors →Increase	
8	Solar Investment Tax Credit	Businesses (Commercial Sectors)	Compensation, Prices, and Costs Block →Production Cost of Individual Industrial and Commercial Sectors →Decrease	Positive
		Households (Residential Sector)	Output and Demand Block →Consumption Reallocation (amount) →All Consumption Sectors →Increase	
9	Energy Demand Decrease from the Energy Supply Sectors		Output and Demand Block →Exogenous Final Demand (amount) for Electric Power Generation, Transmission, and Distribution sector and Natural Gas Distribution sector→Decrease	Negative
10	Water Demand Decrease from the Water Supply Sector		Output and Demand Block →Exogenous Final Demand (amount) for Water, Sewage and Other Systems sector→Decrease	Negative

## 7 Simulation results

In the Base Case, we utilize our best estimates of key input variables. For example, these include the average BCRs for earthquake improvements and average insurance savings. They also include our assumption that property owners will repay their financing by displacing their other spending by an equivalent amount. Also, we do not include offset effects of reduced electricity and natural gas demand for the investment displacement in the Base Case analyses. Various adjustments of all of these inputs and assumptions are made in the sensitivity tests below.

### 7.1 Impacts of California Ygrene PACE financing

Table 3 presents the macroeconomic impacts of the PACE financed improvements on the California economy for both residential and commercial properties for key years over the analysis period (see Rose and Wei, 2019; Appendix E for detailed results for each year from 2013, the year that Ygrene started providing PACE financing, to 2067, the end year of the improvements implemented in 2018 that have a useful life of 50 years). The impacts include employment, gross state product (GSP), gross output (sales revenue), personal income, and non-market value of electricity generated from solar energy. The results are presented in terms of both dollar impacts and percentage changes from baseline. For GSP, gross output, personal income, and non-market value of electricity generated from solar energy, the Net Present Values (NPVs, at a 5% rate of discount) over the entire analysis period are also presented.

**Table 3:** REMI simulation results for the base case: Macroeconomic impacts on California of Ygrene PACE financing for key years.

**Differences from Baseline Level**													

**Variable**	**Units**	**2013**	**2014**	**2015**	**2016**	**2017**	**2018**	**2028**	**2038**	**2048**	**2058**	**2067**	**NPV (or Total Person-Year Jobs)**

Total Employment	Individual (Jobs)	17	179	1,102	3,326	2,483	723	−5	170	50	76	24	9,774^a^
Gross Domestic Product	Mil of Fixed (2015) $	1.62	17.31	109.25	337.03	259.60	83.32	0.69	18.18	3.57	9.19	2.86	661.44
Output (Sales Revenue)	Mil of Fixed (2015) $	3.17	33.97	214.96	661.42	512.69	160.97	−0.56	34.57	6.79	18.58	5.79	1,279.15
Personal Income	Mil of Fixed (2015) $	1.09	11.46	70.34	212.71	142.62	41.76	5.34	14.98	8.77	16.56	5.16	490.50
Non-Market Electricity Production	Mil of Fixed (2015) $	0.07	0.64	3.21	12.76	17.58	19.50	19.59	19.77	0.00	0.00	0.00	257.53

**Percent Change from Baseline Level**													
**Variable**	**Units**	**2013**	**2014**	**2015**	**2016**	**2017**	**2018**	**2028**	**2038**	**2048**	**2058**	**2067**	

Total Employment	Individual (Jobs)	0.0001%	0.0008%	0.0044%	0.0135%	0.0102%	0.0030%	0.0000%	0.0006%	0.0002%	0.0003%	0.0001%	
Gross Domestic Product	Mil of Fixed (2015) $	0.0001%	0.0006%	0.0041%	0.0125%	0.0095%	0.0031%	0.0000%	0.0004%	0.0000%	0.0001%	0.0000%	
Output (Sales Revenue)	Mil of Fixed (2015) $	0.0001%	0.0008%	0.0043%	0.0132%	0.0100%	0.0031%	0.0000%	0.0004%	0.0000%	0.0001%	0.0000%	
Personal Income	Mil of Fixed (2015) $	0.0000%	0.0006%	0.0030%	0.0089%	0.0060%	0.0018%	0.0002%	0.0004%	0.0002%	0.0003%	0.0001%	

a Represents the total cumulative person-year of jobs over the entire analysis period, rather than sustained jobs over the years.

The results indicate that, during the up-front investment period (2013–2018) of the Ygrene PACE financed property improvements, an average annual increase of GSP of $134.7 million and employment of 1,305 jobs^9^ result from the aggregate stimulus effects from the expenditure of the PACE financing. The aggregate GSP and employment impacts become negative from 2019 to 2027, primarily because the negative impacts from the repayment of the PACE financing by the property owners exceed the positive impacts from utility bill savings. From 2028 to 2067, the GSP and employment impacts become positive again, as the period of many PACE financing repayments phase out (the repayment period is usually between 10 and 30 years), and the lasting positive impacts from utility bill savings lead to positive aggregate GSP impacts. The NPV of GSP impacts over the entire period (2013–2067) is estimated to be $661.4 million, despite the negative impacts during the years of 2019–2027. The total cumulative person-year jobs generated are 9,774. The NPVs of the gross output impacts, personal income impacts, and non-market value of electricity generation are estimated to be $1,279.15 million, $490.5 million, and $257.53 million respectively.^10^

We also performed decomposition analyses for the GSP and employment impacts to evaluate how the various economic factors affect the aggregate macroeconomic results.

**Fig. 1:**
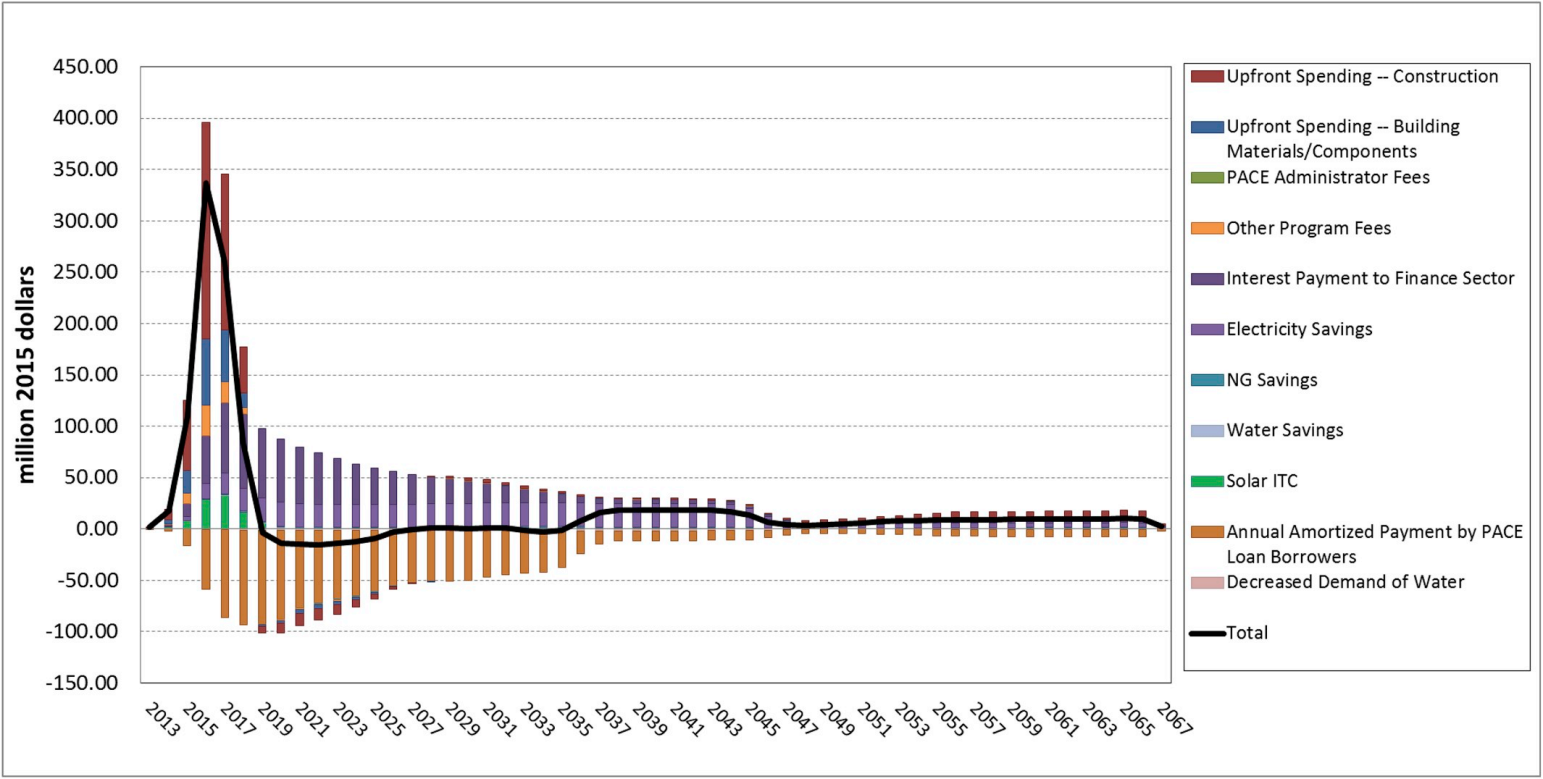
GSP impacts for California of Ygrene PACE Financing (million 2015$).

Fig. 1 depicts the decomposed effects in terms of GSP impacts for all the impact components. The bars in different colors represent impacts of individual stimuli and individual dampening effects. The black solid line indicates the total net impact. The results indicate that the investment expenditures in Construction and Materials/Components Manufacturing sectors result in the highest positive impacts on the state economy in the investment period (2013–2018), and the energy bill (electricity and natural gas) savings result in the highest positive impacts on the economy over the entire analysis period. The PACE financing repayment results in the highest negative impacts, which is only partially offset by the stimulus effects to the Finance sector.

Note that the positive impacts of the non-market production of electricity can be added to REMI Model results for Gross Output (Sales Revenue) to obtain an “Augmented Gross Output” result. Analogous “Augmented GDP” and “Augmented Personal Income” can be estimated by applying value-added or personal income coefficient of the electric utility sector to the “Augmented Gross Output” result. Note, however, that the non-market electricity production does not generate any employment.

### 7.2 Summary of Base Case regional macroeconomic impact results

Table 4 presents a summary of the various impacts of Ygrene residential and commercial PACE financing for California. In the first partition of the table, “Direct Benefits” of PACE financing are presented. In the second partition, the Regional (State) Macroeconomic Benefits (“co-benefits”) stemming from various positive and negative stimuli associated with PACE financing are presented. The decomposed economic impacts are presented in the first and second numerical columns in terms of GDP and employment impacts. Water and energy savings and GHG reduction impacts (all in physical quantities) are presented in the last column of the table. GHG emission reductions are also translated into GDP impacts by applying the social cost of carbon. Note, however, that not all of these direct benefits can be included in the bottom line GDP impacts. Strictly speaking GDP includes only the value of all final goods and services bought and sold in the marketplace. Hence, the “Non-Market Electricity Production” cannot be included, nor can the dollar value of “Greenhouse Gas Reductions”. However, both of these can be included in a measure of “Augmented GDP” (AGDP), an increasingly used measure of aggregate well-being in the vein of “green accounting”. In addition, GDP only includes the “flow” of goods and services, and “Avoided Disaster Losses (in terms of property damage)" (the dollar value of which is presented in brackets) refers to a change in the “stock” of assets, which are not part of GDP; hence, they are not included even in AGDP. The reader is encouraged, however, to keep the avoided property damage in mind as another co-benefit of PACE financing. Finally, we do include the “Avoided Disaster Losses (in terms of relocation costs)" in the AGDP total.

**Table 4:** Summary of Ygrene PACE financing impacts in California (base case).

Type of Impacts	GDP Impacts^a^ (million 2015$)	Employment Impacts^b^ (person-year jobs)	Energy, Water, and Environmental Impacts
**Direct Benefits Stemming from:**			
Water Consumption Reduction (billions of gallons)	(9.16)^c^	87^d^	2.36
Electricity Consumption Reductions (million MWh)	(196.37)^c^	836^d^	3.63
Natural Gas Consumption Reductions (bcf)	(9.25)^c^	83^d^	2.86
Non-Market Electricity Production (million 2015$)	131.66^e^	n/a^f^	n/a
Greenhouse Gas Reductions (metric MtCO2e)	54.53^g^	n/a	1.15
Avoided Disaster Losses (property damage)^h^	[2.36]^i^	n/a^j^	n/a
Avoided Disaster Losses (relocation costs)	0.38^k^	n/a^j^	n/a
**Regional Macroeconomic Benefits Stemming from:**			
Ygrene Financing for Improvements	491.29	7,010	n/a
Ygrene and Other Program Fees	54.08	772	n/a
Interest Payments for Financing	486.98	7,019	n/a
Water Cost Savings	7.86	174	n/a
Energy (Electricity and Natural Gas) Cost Savings	320.74	6,841	n/a
Solar Investment Tax Credit	76.41	1,103	n/a
Annual Repayment of PACE Financing	−754.85	−12,744	n/a
Decreased Demand from Water Supply Sector	−21.09	−402	n/a
Total GDP	661.42	9,773	n/a^l^
Total AGDP	847.99	9,773	n/a^l^

a Net present value (NPV) at a 5% discount rate over the period 2013–67.

b Total new jobs created over the period 2013–67.

c Calculated by first applying projected state average price to energy/water savings in physical terms to obtain estimates in gross output changes and then by applying value-added to gross output ratios to obtain changes in GDP; not included in Total Impacts to avoid double-counting, because its direct and indirect effects are included in the Regional Macroeconomic Benefits partition.

d Calculated by applying employment coefficients to gross output estimates; not included in Total Impacts to avoid double-counting.

e The non-market value of electricity production from solar energy is $257.53 million. This is converted to GDP by using the value-added to gross output ratio in the electricity sector (the average of the ratios in the private and government electricity sectors). This dollar amount is not included in Total GDP, but is included in Total AGDP.

f There are no direct employment impacts of non-market electricity production over and above the cost of installation of solar generation capability, which is captured in the Ygrene Financing row below.

g Based on NRC (2017) estimate of $42 per ton of CO_2_ (in 2007$ and converted to $47.57 in 2015$). This dollar amount is not included in Total GDP, but is included in Total AGDP.

h Also includes a very small amount of prevented business interruption in commercial facilities.

i Pertains to the prevention of property damage primarily for residences; hence, not included in GDP or AGDP Total.

j We did not have sufficient data to estimate this impact.

k This dollar amount is not included in Total GDP, but is included in Total AGDP.

l The total cannot be computed for this column because entries are in different units of measure.

As our focus is on estimating the macroeconomic co-benefits of PACE financing, we only provide a brief summary of the direct benefits: reductions in water and energy consumption, in GHG emissions, and in losses from natural disasters. It is important to note that these reductions are brought about by financing that basically pays for itself in terms of cost savings on utility bills and avoided costs from the need to repair homes and commercial buildings. The reader is referred to the first partition of Table 4, which refers to these “Direct Benefits”. Non-market benefits again are not counted in the Total GDP but are counted in Total AGDP. Note also that those direct benefit numbers presented in parentheses in the first partition are not added to Total GDP or AGDP at the bottom of the tables to avoid “double-counting” because not only their direct, but also their indirect, impacts are factored in the second partition, as calculated by the REMI Model (e.g., “Energy Cost Savings”). The direct impacts in California are sizeable, amounting to about $400 million (not including a few million dollars in benefits relating to avoided property damage).

**Table 5:** Changes in GHG emissions and energy and water consumptions in California due to Ygrene PACE improvements.

	Baseline Values (2013–67)	Reductions Due Directly to Ygrene PACE Improvements (2013–67)		Increases (Offsets) Due to Stimulated Economic Activities (2013–67)		
		level	percent	level	percent of reductions	percent of baseline
GHG Emissions (MMCO2e)	11,124	1.15	0.0103%	0.144	12.5%	0.0013%
Electricity Consumption (million MWh)	14,735	3.63	0.0246%	0.099	2.7%	0.0007%
NG Consumption (bcf)	130,842	2.86	0.0022%	0.840	29.4%	0.0006%
Water Use (billions of gallons)	572,193	2.36	0.0004%	3.977	168.5%	0.0007%

Total GDP impacts (in net present values) are $661.4 million for California. The employment impacts are 9,774 person-year jobs. The largest contributor to these impacts emanate from Energy Cost Savings, followed by the net impacts of Ygrene Financing, Interest Payments and Other Program Fees minus Annual Repayment of this Financing.

Total AGDP impacts (including non-market value of solar electricity production, social cost of carbon, and avoided disaster relocation cost) are nearly $850 million. The value of Non-Market Electricity Production becomes the third largest contributor to these impacts.

### 7.3 Offsetting effects

In evaluating the contribution of PACE financing to reduce GHG emissions, natural resource use and hazard reduction, it is also important to consider any offsetting effects. Ironically, the same economic stimuli that are the focus of this report have the potential to engender this “offset” effect, in that increases in economic activity generate additional GHGs and utilize more water, natural gas and electricity. The increased amounts emanate from direct effects in the production of inputs into building improvements and the spending of workers who receive income from producing and installing the improvements. They also emanate from the supply-chain effects of production and further consumer spending of additional income generated, as well as consumer spending from energy and water bill savings and reduced costs of natural disasters.

Table 5 presents the results of our analysis of these offset effects. The results were estimated by utilizing gross output increases by year multiplied by historical and projected emission and resource use factors derived from several sources.^11^

Note that the offset effects for GHGs and electricity are a minimal 12.5% and 2.7%, respectively. However, the offset is a significant 29.4% for natural gas and much more than offsets the direct reductions in the case of water. Note, however, the offset effect represents only 7 ten-thousandths of a percent of baseline water use in the state.

Overall, as with regard to most policy improvements, there are some offsetting effects in relation to stated objectives and/or negative side-effects in other domains. Policymakers must weigh these trade-offs in evaluating the policies. In the case of Ygrene financing, the net environmental gains are still positive in three of the four cases. For the remaining case of net increases in water use, it should be noted that those offsets are larger than the savings because of the small percentage of water conservation projects in the state.

## 8 Sensitivity analyses

We perform several sensitivity tests to analyze how the changes in some key assumptions would affect the macroeconomic impact analysis results of the PACE financing.

### 8.1 Displacement effects of repaying PACE financing

We first perform a sensitivity test on our assumption that people need to reduce other purchases to repay PACE financing on a dollar-for-dollar basis. It is possible that they will dip into savings, so as not to fully displace other spending. We therefore performed a sensitivity test that reduces the direct offset by 10%. The GDP and employment impacts are increased by 11.4% and 13.0%, respectively, indicating that the Base Case results are not very sensitive to this assumption.

### 8.2 Offsetting effects of energy demand reduction

We also include the dampening impacts from the decreased demand for electricity and natural gas from the energy supply sectors. These impacts are simulated as decreases in exogenous final demand from the Electricity Generation sector and Oil and Gas Extraction sector in the REMI simulations.

The primary reason for excluding this offset in the Base Case is that California law (under Senate Bill 350) requires the state to increase the share of electricity derived from renewable sources from 33% to 50% and to double the efficiency of existing buildings. Additionally, the California Energy Commission (CEC) established the Existing Buildings Energy Efficiency Action Plan to increase energy efficiency in residential, commercial, and government buildings. All of these policies and regulations are entirely independent of PACE, and most of them pre-dated the establishment of PACE programs in these states. Thus, PACE only serves to assist in achieving efficiency and renewable targets that are already mandated by state law and Public Utility Commission regulations. However, it is possible renewable energy and efficiency targets will more than be met by responses to market conditions, in which case PACE displacements of electricity and natural gas would represent an overage and justify including their value in the reduction of electricity and natural gas production. In our sensitivity test, we have thus assumed a 50% offset.

Under this offset assumption, the Base Case GDP impacts are reduced from $661.4 million to $482.7 million. Thus, the estimated economic impacts are somewhat sensitive to the inclusion of the offsetting effects of energy demand reduction. This is because over 92% of the Ygrene PACE improvement investment in California is energy efficiency upgrades and renewable energy installations. The esimates for California are most sensitive to this assumption, resulting in a reduction of Base Case GDP Impacts by 27%.

### 8.3 Additivity of Ygrene Financing

A question arises as to the extent to which the various improvements simulated here would have taken place in the absence of Ygrene financing, i.e., whether the impacts are truly additive. Estimates by Kirkpatrick and Bennear (2014) indicate 100% additivity for PACE financing in general on solar energy improvements, and more recent estimates by Eyer (2019) indicate 25%–100% additivity for solar energy. If we consider the Eyer estimate to represent a lower-bound not just for solar energy improvements but all improvements, we would simply reduce the Base Case estimates by 75%. This will reduce the Base Case GDP impacts from $661.4 million to $165.36 million.

## 9 Conclusions and policy implications

### 9.1 Policy implications

PACE financing, like many other government or government-sanctioned programs, has been justified not only by the direct benefits it would generate but also by various co-benefits, one of them being their potential to stimulate the economy. It cannot be taken for granted that positive net economic benefits are forthcoming for every new program, and a careful study of economic impacts, such as that presented above, is warranted. It is then up to policymakers in their program evaluation to determine how much weight to give to these co-benefits. One approach is to juxtapose them along with the direct benefits, of course, to any downsides of the policy/program.

PACE financing has not come without criticism. The most significant opposition comes from the Federal Housing Finance Agency (FHFA) concerning the PACE assessment's super lien status, opposition shared by the Mortgage Bankers Association and the National Association of Realtors. PACE financing is voluntarily applied to the property as a special assessment added to the property tax bill, and as such, like all property taxes, is senior to any first mortgage, and thus in the event of foreclosure, only the portion of the PACE assessments that are due at that point in time are paid out before the mortgage. In 2010, the FHFA, as conservator of government sponsored enterprises (GSEs) Fannie Mae and Freddie Mac following the 2008 financial crisis, instituted policy that Fannie and Freddie would not purchase or refinance a mortgage with a PACE assessment. This policy was based on the objection that PACE assessments added risk to Fannie and Freddie's assets by displacing them as prime mortgage holders in the event of property owner default and foreclosure. It is important to note, however, that because PACE is a voluntary special assessment, and cannot be fully accelerated by the taxing jurisdiction, the PACE administrator, or any entity other than the property owner, only the annual PACE assessments in arrears would be due in the event of foreclosure. Thus, the risk to Fannie and Freddie as a result of PACE is not the entire PACE obligation itself, but only the annual assessments past due. Typically, PACE assessments cannot exceed 10–15% of the fair market value of the property, and the combined loan-to-value ratio of PACE and the underlying mortgage cannot exceed 90–100% depending on the state's PACE statute.^12^ In the case of Ygrene, the average PACE assessment loan-to-value ratio is 7.06% and the average combined (mortgage balance plus PACE assessment) loan-to-value ratio is 59.05%, both well below the statutory maximums. Moreover, it appears that the number of reported mortgages that have PACE assessments is rather small. In addition, analysts have found that PACE improvements increased house values by at least the cost of the financing (Goodman and Zhu, 2016).

In December of 2017, the Federal Housing Agency (FHA) issued Mortgagee Letter 2017–18, joining FHFA's opposition to PACE, instituting a policy that it would not insure any new mortgages with a PACE assessment, reversing its previous position put in place in 2016 that it did insure mortgages with PACE assessments. The FHA cited similar reasons as FHFA for its objection to PACE. However, the California Alternative Energy and Advanced Transportation Financing Authority (CAEATFA) established a State-funded loan loss reserve program in 2010 that provided first mortgage holders the ability to recover any losses as a result of residential PACE in the event of foreclosure. Of course, one solution, should default issues accelerate (Grind, 2017), would be for PACE finance companies to take a second position on mortgages that still have the assessment remained in the event of the sale, refinancing or foreclosure (Goodman and Zhu, 2016). However, taking a second position is a drastic step and goes against the grain of property tax assessment, a unique feature of PACE financing that provides many benefits. Such a step would significantly reduce the availability of PACE financing as an option in the market, as well as driving up the cost of capital, and thus could possibly eliminate PACE altogether.^13^

FHFA and, to a lesser extent, FHA policy opposition toward PACE is one of the most significant barriers to the expansion of residential PACE enabling legislation and broader PACE market expansion. Initial PACE legislation was established in California in 2008. Soon after California, many other states enacted PACE legislation. Today, thirty-six states, as well as Washington DC, have enacted PACE legislation, twenty of which, and Washington DC, have active commercial PACE programs. Yet, only three states – California, Florida, and Missouri – have active residential and commercial PACE programs operating simultaneously.

Additionally, there has been some criticism from individuals and consumer protection organizations arguing that PACE financing lacks regulation and needs stricter guard rails to protect consumers from potential abuse from unprofessional contractors and misunderstandings about how PACE works. California has addressed many of these concerns through recent legislation^14^ requiring residential PACE administrators to provide financial disclosure forms (similar to “Know Before You Owe” language in the mortgage industry), requiring “confirm terms” calls to the property owners to review all financing terms and information, as well as tightening underwriting criteria and adding an ability-to-pay threshold, where applicable, among others. Assembly Bill 1284, passed in 2017, also tasked the California Department of Business Oversight (DBO) as the statewide regulator of PACE administrators. The administrators are also regulated by the hundreds of city and county boards that enable PACE programs through establishment of guidelines and rules for the programs themselves, as well as the operations of PACE administrators. This regulatory apparatus is much different than that of other financing industries that are typically regulated by only a few regulatory bodies at either the State or Federal level. It is also important to note that the California State Treasurers Office found there was a significant drop in total PACE activity in California after the new legislation took effect (California Alternative Energy and Advanced Transportation Financing Authority (CAEATFA), 2018).

California's recent legislation, FHFA and FHA policy toward PACE, and broader banking and realtor opposition to PACE have, and will continue to have, significant influence on the future of the national, state, and local PACE markets. As state legislators, federal regulators, and policy makers consider how to approach PACE policy, we intend that our research will play a role in how that policy takes shape. For example, future PACE policy could have a significant impact on whether California will meet its GHG emission reduction goals under Assembly Bill 32 passed in 2006, its goal to double the energy efficiency of existing buildings by 2050 under Senate Bill 350 passed in 2015, and, most recently, its goal to achieve 100% renewable energy by 2045 under Senate Bill 100 passed in 2018.

As this research shows, PACE programs can have significant positive impacts on GDP, job creation, energy and water conservation, natural hazard mitigation, and greenhouse gas emission reduction, among others. These positive impacts exceed any negative impacts measured to date.

### 9.2 Conclusion

The Property Assessed Clean Energy (PACE) Program has broadened significantly in recent years and now also includes financing for saving water and for reducing vulnerability to disasters for both residential and commercial properties. It is able to achieve its direct societal objectives while providing financial gains to those receiving financing and implementing the building improvements. It does so by providing financing that is beneficial to the recipients by saving money on utility bills and avoiding having to pay for building repairs or reconstruction following an earthquake. Moreover, it represents an equitable alternative collateralization of the financing, in part because the property value is not a criterion for qualifying nor is a credit score, thereby making it available to some who could not otherwise secure financing through more conventional lending-related instruments.

We have estimated the direct benefits of PACE financing by one of its major administrators, Ygrene Energy Fund, Inc. More uniquely, we have also estimated the broader macroeconomic co-benefits of this financing in terms of impacts on market-based GDP and augmented GDP that takes into account the non-market effects. The analysis was performed in the context of an Economic Consequence Analysis that took into consideration the numerous positive and negative stimuli associated with PACE financing. The macro impacts were estimated with the use of the REMI Policy Insight Plus Model, the most widely used macroeconometric model at the state and local levels in the US.

Total Augmented GDP (AGDP) impacts, which include the non-market value of solar electricity production, social cost of carbon, and avoided disaster relocation cost, are nearly $850 million for California. Employment impacts are the same as the regular employment impacts noted above, since new jobs are not directly associated with any of these direct environmental and hazard reduction benefits.

Sensitivity tests indicate our results are robust to changes in major assumptions relating to displacement effects of repaying PACE financing, offsetting effects of energy demand reduction, alternative estimates of insurance savings, and the relative additivity of Ygrene financing. Overall, the PACE financing provided by Ygrene, and even more so if we included all PACE financing, generates sizable net positive impacts on the economies of the major states of operation.

PACE is a public policy tool designed to leverage private capital to help homeowners and business owners overcome barriers to implementing building improvements and to provide broader public benefits. PACE financing has successfully reduced air pollution emissions and resource use but has faced criticism by competitors and some analysts because it is not traditional in terms of collateral and because of some marketing and quality control issues of a small percentage of independent contractors that implement the various improvements. The industry addresses this through compliance guidelines for each PACE program. In addition to the more straightforward direct benefits of PACE financing, this paper provides estimates of the several economic *co-benefits*, which are very positive on net. These co-benefits are thus also likely to be of considerable interest to policy-makers evaluating future decisions about accommodating and even facilitating PACE financing in addition to the broader public policy goals of achieving federal, state, and local energy, environmental, and disaster mitigation goals.

PACE is a financing mechanism by which broader and more equitable access to property improvements in energy efficiency, water conservation, renewable energy, and hazard mitigation is made possible. This research shows that PACE has had a positive net impact on the environment and the economy in one of the major states in which it is enabled.

## Declaration of competing interest

The authors declare that they have no known competing financial interests or personal relationships that could have appeared to influence the work reported in this paper.

## Figures and Tables

**Table 1:** Data, Refinement and Sources

Data Type	Description	Coverage	Source	Assumptions/Modifications	Comments
1a. PACE Financing	major assessment characteristics	# of PACE improvements: CA: 32,513 FL: 21,855 MO: 162	Ygrene	--a	–

1 b. Useful Life of Improvement	period generating benefits	all assessments	Ygrene	varies by improvement type	–

1c. Interest Rate	interest rate on assessments	all assessments	Ygrene	varies by state & year & project	–

1 d. Fees	paid to Ygrene & governments	all assessments	Ygrene	varies by state & year & project	–

2. Production of Improvements	mapping improvement expenditures to REMI sectors	160 REMI sectors	NAICS website; specific expenditure references	–	–

3. Material/Labor Share of Improvements	disaggregation of expenditures	all assessments	various improvement type websites	labor part of Construction & Repair sector^b^	–

4a. Energy Savings (expenditures)	fossil energy reduction; overall energy reduction	all assessments (annual)	Ygrene	–	–

4 b. Energy Savings (quantity)	fossil energy reduction; overall energy reduction	all assessments (annual)	Ygrene	–	–

4c. Energy Savings (value)	value of energy saved	all assessments (annual)	Ygrene	valued at market price^c^	–

4 d. Energy Produced (solar electricity quantity)	quantity used + quantity sold to the grid	all assessments (annual)	Ygrene	valued at market price	–

4e. Energy Production Displaced (quantity)	fossil fuel demand reduction; same as Energy Savings quantity	quantity offset by solar & energy efficiency	Ygrene	–	calculated from Ygrene data

5a. Water Savings (quantity)	decrease water utilization	all assessments (annual)	Ygrene	–	–
					
5 b. Water Savings (value)	decrease water utilization	all assessments (annual)	Ygrene	valued at market price^c^	–

5c. Water Production Displaced (quantity)	same as Water Savings quantity	all assessments (annual)	Ygrene	–	calculated from Ygrene data

5 d. GHG Damage Avoided 6a. Hazard Mitigation Improvements (expenditures)	social cost of carbon expenditure on hazard risk reduction	carbon but not methane all assessments (annual)	NRC (2017) Ygrene	– –	– –

6 b. Hazard Mitigation Savings (value)	Dollar value of losses avoided	all assessments (annual)	*Mitigation Saves 2* benefit-cost ratios	apply BCRs to expenditures;^c^ adapt MMC & IBHS data	–

6c. Insurance Savings	premium reduction	all assessments (annual)		–	

a See Rose and Wei (2019) for minor adjustments.

b Minor exceptions, such as Landscaping Services sector for drought-resistant landscaping improvements.

c Value saved assumed to be spent entirely on consumer goods by home owners and to reduce production costs for owners of commercial property.

## Footnotes

1 For a discussion of various other pros and cons of PACE financing, we refer the reader to Pozdena and Josephson (2011); Sichtermann (2011); Goodman and Zhu (2016); RMI (2017); DBRS (2018); Deason and Murphy (2018).

2 During this period Ygrene also financed 21,760 residential properties and 89 commercial properties in Florida and 162 residential projects in Missouri.

3 We are not able to estimate the amount of methane emissions from the extraction, storage or transportation of natural gas.

4 Labor services are embedded in the production of materials and equipment but are separately calculated for the installation activities.

5 Residential Baseline Energy Consumption was obtained from the Energy Information Administration's Residential Energy Consumption Survey (EIA, 2018a; EIA, 2018b), and varies by state, household income bracket, and number of household members; Commercial Baseline Energy Consumption was obtained from the Energy Star Portfolio Manager, U.S. Energy Use Intensity by Property Type (EPA and DOE, 2018), and varies by building type, and square footage.

6 For Example: Lighting, HVAC, Building Envelope – Insulation, Building Envelope – Cool Roof, Solar PV, Water Efficiency – Toilets/Showers/Faucets, Water Efficiency – Hardscape and Artificial Turf, etc.

7 Note that the energy and water savings calculated are engineering level estimates (deemed savings) rather than actual savings determined at the meter or property-level.

8 GHG emission factor for electricity is 0.2726 MtCO2e/MWh in CA and 0.5345 MtCO2e/MWh in FL (EPA, 2017). The GHG emission factor for natural gas combustion is 54.5 kgCO2e per mcf (EPA, 2014). These GHG emission factors include CO_2_, CH_4_ and N_2_O, and are converted to a CO_2_ equivalent based on the 100-year global warming potential of CH_4_ and N_2_O.

9 A job is defined as a person-year of employment full-time equivalent. Results presented for a given year represent the jobs in place that year whether they are new jobs or carryovers from past years.

10 Economic activities stimulated by the PACE financing also generate tax revenues to various levels of government. We also estimated the direct and indirect tax revenues based on the REMI macroeconomic impact results and tax data obtained from the social accounting matrix (SAM) for California (IMPLAN, 2018). The net present value (NPV) of total tax impacts is estimated to be $120.9 million, with personal income taxes accounting for about 65% of the total, and indirect business taxes and corporate income taxes accounting for 17.5% each. About 45% of the increased tax revenues go to state/local governments, and 55% go to the federal government.

11 The sources of data for baseline values for the environmental variables are: U.S. Energy information Administration (EIA), 2018e, U.S. Energy information Administration (EIA), 2018d, U.S. Energy information Administration (EIA), 2018c; USGS (2018); California Air Resources Board (CARB) (2018); California Department of Water Resources (CDWR) (2014).

12 PACE loan-to-value and total combined loan-to-value thresholds vary by state and in some cases property type (residential, commercial, etc.). However, most active PACE programs have limits on the size of the PACE assessment in relation to the fair market value of the property, which are either established by state statute and/or the PACE program guidelines and regulations established by local jurisdictions. For example, under the California Financial Code, Division 9, Chapter 3.5, Section 22,684, the PACE financing amount must be less than 15% of the value of the property, up to the first seven hundred thousand dollars and less than 10% of the remaining value, and the total PACE assessments and mortgage-related debt combined must not exceed 97% of the market value.

13 Of the few states that have subordination clauses in their PACE enabling legislation, not one of those states has an active or established residential PACE program (e.g., Vermont and Oklahoma).

14 California Assembly Bill 2693, 2016; California Senate Bill 242, 2017; and California Assembly Bill 1284, 2017.
